# Approximation to Hadamard Derivative via the Finite Part Integral

**DOI:** 10.3390/e20120983

**Published:** 2018-12-18

**Authors:** Chuntao Yin, Changpin Li, Qinsheng Bi

**Affiliations:** 1 Department of Mathematics, Shanghai University, Shanghai 200444, China; 2 Faculty of Civil Engineering and Mechanics, Jiangsu University, Zhenjiang 050043, China

**Keywords:** Hadamard derivative, finite part integral

## Abstract

In 1923, Hadamard encountered a class of integrals with strong singularities when using a particular Green’s function to solve the cylindrical wave equation. He ignored the infinite parts of such integrals after integrating by parts. Such an idea is very practical and useful in many physical models, e.g., the crack problems of both planar and three-dimensional elasticities. In this paper, we present the rectangular and trapezoidal formulas to approximate the Hadamard derivative by the idea of the finite part integral. Then, we apply the proposed numerical methods to the differential equation with the Hadamard derivative. Finally, several numerical examples are displayed to show the effectiveness of the basic idea and technique.

## 1. Introduction

During the last several decades, many efforts have been made in the study of fractional calculus and entropy to investigate the dynamical behavior [1,2,3,4,5,6]. Entropy is often regarded as a crucial index to describe the statistical characteristics in complex systems. Beyond the complexity appearing in complex systems, the fractionality emerging in fractional dynamical systems has gradually attracted interest. Entropy, having an important role in exploring complexity, has been further developed to disclose fractionality in fractional differential systems in [5], where the author presented a novel expression for entropy with the aid of the properties of fractional calculus. Besides, the authors in [6] analyzed the complexity of the self-excited and hidden chaotic attractors in a fractional-order chaotic system by computing their spectral entropy and Brownian-like motions.

Fractional calculus has appeared extensively in a variety of realms, such as in physics, mechanics, dynamics, engineering, finance, and biology [7,8,9,10,11,12]. Up to now, there exist many kinds of fractional integrals and derivatives like Riemann–Liouville, Caputo, Riesz, Grünwald–Letnikov, and Hadamard integrals and derivatives. However, it has been noticed that most of the work is devoted to the issues related to Riemann–Liouville, Caputo, and Riesz derivatives [13,14]. Actually, the Hadamard derivative is also very worthy of in-depth study. There are two differences between the Hadamard derivative and the Riemann–Liouville one. To be specific, the basis function of the integral appearing in the Hadamard derivative is in the logarithmic form (logx−logt), but the basis function takes the form (x−t) in the Riemann–Liouville one. On the other hand, the Hadamard derivative is viewed as a generalization of the operator ${\left(x\frac{d}{dx}\right)}^{n}$, while the Riemann–Liouville derivative is considered as an extension of the classical differential operator ${\left(\frac{d}{dx}\right)}^{n}$. Such distinguishing features of the Hadamard derivative make it extensively used in many problems related to mechanics and engineering, e.g., the fracture analysis of both planar and three-dimensional elasticities. For more details about Hadamard fractional derivatives and integrals, the reader can refer to the studies [15,16,17,18,19] and the references therein. Particularly deserving of mention, Kilbas investigated the fractional integration and differentiation in the frame of the Hadamard setting [17]. Furthermore, the authors in [18] studied the Mellin transform of Hadamard fractional calculus, and the integration by parts for the Hadamard-type integral was shown, as well. In [19], Ma and Li studied the fundamental properties of Hadamard fractional calculus and proposed the well-posed conditions for the fractional differential equation with the Hadamard derivative. For some further studies on the Hadamard integral and/or derivative, see [20,21,22,23].

There exist some papers about the analysis of fractional entropy [24,25]. In particular, the authors in [25] devoted their work to the fractional-order entropy analysis of earthquake data series. It is worth noting that investigations related to the entropy analysis of earthquakes are of great significance to human beings. We know that the fracture phenomena will appear when earthquakes happen. As mentioned earlier, the Hadamard integral and derivative often arise in the formulation of fracture analysis. Additionally, in the scope of statistical mechanics, entropy is a logarithmic measure of the number of states with a significant probability. In [26], the authors investigated Hadamard fractional differential equations with varying coefficients in the probability sense. The Hadamard derivative is a nonlocal fractional derivative with a singular logarithmic kernel with memory; hence, it is suitable to describe complex systems. For these reasons, the study of the Hadamard derivative is necessary and useful for the entropy analysis. In this paper, we devote our work to the evaluation of the Hadamard derivative. From the definition of the Hadamard integral, we note that it is difficult to get the exact analytical expression of a given function, the same as for the classical integral. Therefore, it is often necessary to obtain its approximation value. Dating back to 1923, Hadamard encountered a class of integrals with strong singularities when using a particular Green’s function to solve the cylindrical wave equation. He ignored the infinite parts of such integrals after integrating by parts. In doing this, the values of the integrals can be calculated. Such an idea is very significant and practical and can be used directly in many physical models, such as the crack problems of both planar and three-dimensional elasticities. Diethelm gave an implicit algorithm for the approximate solution of the fractional differential equation with the Riemann–Liouville derivative in the sense of the finite part integral in [27]. Recently, Ma and Li derived an expression of the Hadamard derivative by using the finite part integral [28]. There also exist some works on the numerical calculation using the finite part integral [29,30,31,32]. Inspired by such ideas, we construct methods to calculate the Hadamard derivative by employing the finite part integral where the methods are based on the fractional rectangular formula and the fractional trapezoidal one. Additionally, we apply the derived methods to solve the fractional differential equation with the Hadamard derivative, as well.

The outline of this paper is organized as follows. After introducing some basic concepts and properties about Hadamard fractional calculus in Section 2, the numerical schemes for the Hadamard derivative with order 0<α<1 using the finite part integral are derived in Section 3. Furthermore, we apply the proposed methods to solve the fractional differential equation with the Hadamard derivative in Section 4. In Section 5, we display several numerical examples to verify the usability of the derived approaches. Finally, the last section summarizes this paper.

## 2. Preliminaries

In this section, we recall some fundamental definitions about the Hadamard integral and derivative, and we introduce some properties that can be used thereafter. Let f(x) be a function defined on (a,b), where 0≤a<b≤∞.

**Definition** **1.** *The Gamma function is defined as [1,2]:*

(1) $$\Gamma \left(\alpha \right)=\int_{0}^{\infty }e^{-t}t^{\alpha -1}dt,\;\alpha >0.$$

**Definition** **2.** *The Hadamard integral of f(x) with order α>0 is defined as [1,17]:*

(2) $$H^{D_{a^{+}}^{-\alpha }}f\left(x\right)=\frac{1}{\Gamma (\alpha )}\int_{a}^{x}{\left(\log\frac{x}{t}\right)}^{-(1-\alpha )}f\left(t\right)\frac{dt}{t},\;x>a.$$

**Definition** **3.** *The Hadamard derivative of f(x) with order α>0 is defined as [1,17]:*

(3) $$H^{D_{a^{+}}^{\alpha }}f\left(x\right)=\delta^{n}\left[H^{D_{a^{+}}^{-(n-\alpha )}}f\left(x\right)\right],\;x>a,$$

*where $\delta =x\frac{d}{dx},\;n-1<\alpha \leq n\in Z^{+}$.*

Then, for the Hadamard differentiation operator $H^{D_{a^{+}}^{\alpha }}\cdot$, we define space $AC_{\delta }^{n}\left[a,\;b\right]$ as [1]:

(4) $$AC_{\delta }^{n}\left[a,\;b\right]=\left\{f:\left[a,\;b\right]\to R\;|\;\delta^{n-1}\left[f\left(x\right)\right]\in AC\left[a,\;b\right]\right\},$$

where AC[a,b] is the set of absolutely-continuous functions. In addition, we introduce the weighted space $C_{\gamma ,\;\log}\left[a,\;b\right]$ given as [1]:

(5) $$C_{\gamma ,\;\log}\left[a,\;b\right]=\left\{f\left(x\right)\;|\;{\left(\log\frac{x}{a}\right)}^{\gamma }f\left(x\right)\in C\left[a,\;b\right]\right\},$$

which is endowed with the norm:

(6) $$\begin{array}{cc} {∥f∥}_{C_{\gamma ,\;\log}}= & ∥{\left(\log\frac{x}{a}\right)}^{\gamma }f\left(x\right)∥{}_{C},\\ = & \max_{x\in [a,\;b]}\left|{\left(\log\frac{x}{a}\right)}^{\gamma }f\left(x\right)\right|,\;0\leq \gamma <1. \end{array}$$

Now, we present several properties about Hadamard integral and derivative.

**Lemma** **1.** *Suppose $f\left(x\right)\in AC_{\delta }^{n}\left[a,\;b\right]$. Then, the Hadamard derivative $H^{D_{a^{+}}^{\alpha }}f\left(x\right)$ can be rewritten in the following form [28]:*

(7) $$H^{D_{a^{+}}^{\alpha }}f\left(x\right)=\frac{1}{\Gamma (-\alpha )}=\;\;\;\;\;\;\;\int_{a}^{x}\frac{f(t)}{{\left(\log\frac{x}{t}\right)}^{\alpha +1}}\frac{dt}{t},$$

*where $n-1\leq \alpha <n\in Z^{+}$ and =∫ means taking the finite part of this singular integral.*

**Lemma** **2.** *If α>0,β>0, and 0<a<b<∞, for the logarithmic functions, the following relations hold [1].*

(8) $$H^{D_{a^{+}}^{-\alpha }}\left[{\left(\log\frac{x}{a}\right)}^{\beta -1}\right]=\frac{\Gamma (\beta )}{\Gamma (\beta +\alpha )}{\left(\log\frac{x}{a}\right)}^{\beta +\alpha -1},$$

(9) $$H^{D_{a^{+}}^{\alpha }}\left[{\left(\log\frac{x}{a}\right)}^{\beta -1}\right]=\frac{\Gamma (\beta )}{\Gamma (\beta -\alpha )}{\left(\log\frac{x}{a}\right)}^{\beta -\alpha -1}.$$

## 3. Approximating the Hadamard Derivative via the Finite Part Integral

Due to the distinguishing features of the Hadamard derivative, it is difficult to approach the derivative directly. In this situation, the method of computation needs to be properly defined. Hence, the finite part integral method is naturally presented for the sake of calculation. In the following, we will give the explicit formulation process and error analysis.

Before we come to the main result, we state some lemmas that will be used later on.

**Lemma** **3.** *Let 1<p<2. Suppose $f\in C^{s}\left[a,\;b\right]$ with p−1<s∈N. On a general interval [a,b], the finite part integral is expressed in the following way [29]:*

(10) $$\begin{array}{cc} =\;\;\;\;\;\;\;\int_{a}^{b}{\left(x-a\right)}^{-p}f\left(x\right)dx & =\sum_{k=0}^{[p]-1}\frac{f^{k}\left(a\right){\left(b-a\right)}^{k+1-p}}{[k+1-p]k!}\\ & +\int_{a}^{b}{\left(x-a\right)}^{-p}R_{[p]-1}\left(x,\;a\right)dx, \end{array}$$

*where:*

(11) $$R_{\mu }\left(x,\;a\right)=\frac{1}{\mu !}\int_{a}^{x}{\left(x-y\right)}^{\mu }f^{\left(\mu +1\right)}\left(y\right)dy$$

*is the remainder of the $\mu^{th}$ degree Taylor expansion polynomial of f at point a. [p] is the largest integer not exceeding p.*

**Lemma** **4.** *Suppose the function $f\in C^{s}\left[a,\;b\right]$. For $d\in N_{0},\;0<\alpha <s\leq d+1$ and α∉N, we have [29]:*

(12) $$\rho_{s}\left(R_{n}\right)=\zeta_{d,\;\alpha ,\;s}n^{\alpha -s}+o\left(n^{\alpha -s}\right)$$

*with the constant $\zeta_{d,\;\alpha ,\;s}>0$. Here, the parameter d is the degree of the compound quadrature formula, and the error constants $\rho_{s}\left(R_{n}\right):=\sup\{|R_{n}\left[f\right]|:f\in C^{s}\left[0,\;1\right]\;and\;∥f^{\left(s\right)}∥\leq 1\}$.*

Now, we show how to get the approximate value of the Hadamard derivative via the finite part integral.

First, we transform the finite part integral of the Hadamard derivative into the standard interval. By means of the change of variables $\log\frac{x}{t}=u\log\frac{x}{a}$ and $t=x{\left(\frac{x}{a}\right)}^{-u}$, we can obtain:

(13) $$\begin{array}{cc} & \frac{1}{\Gamma (-\alpha )}=\;\;\;\;\;\;\;\int_{a}^{x}\frac{f(t)}{{\left(\log\frac{x}{t}\right)}^{\alpha +1}}\frac{dt}{t}\\ = & \frac{1}{\Gamma (-\alpha )}{\left(\log\frac{x}{a}\right)}^{-\alpha }=\;\;\;\;\;\;\;\int_{0}^{1}u^{-(\alpha +1)}g\left(u\right)du, \end{array}$$

where $g\left(u\right)=f\left(x{\left(\frac{x}{a}\right)}^{-u}\right)$.

For an integer *N* and a given *x*, let $a=x_{0}<x_{1}<\cdots <x_{n}<x_{n+1}<\cdots <x_{N}=x$ be a uniform partition of the interval [a,x] with the step $h=x_{n+1}-x_{n}=\frac{x-a}{N},\;n=0,\;1,\;\cdots ,\;N-1$. Correspondingly, the approximate value of the finite part integral of the Hadamard derivative at the point $x_{n}$ is:

(14) $$\begin{array}{cc} & \frac{1}{\Gamma (-\alpha )}=\;\;\;\;\;\;\;\int_{a}^{x_{n}}\frac{f(t)}{{\left(\log\frac{x_{n}}{t}\right)}^{\alpha +1}}\frac{dt}{t}\\ = & \frac{1}{\Gamma (-\alpha )}{\left(\log\frac{x_{n}}{a}\right)}^{-\alpha }=\;\;\;\;\;\;\;\int_{0}^{1}u^{-(\alpha +1)}g\left(u\right)du\\ = & I, \end{array}$$

where $g\left(u\right)=f\left(x_{n}{\left(\frac{x_{n}}{a}\right)}^{-u}\right)$.

Denote $I_{1}==\;\;\;\;\;\;\;\int_{0}^{1}u^{-(\alpha +1)}g\left(u\right)du$. We divide the standard interval [0,1] into $0=u_{0}<u_{1}<\cdots <u_{n}=1$ with the step $h=\frac{\log\frac{x_{n}}{x_{n-1}}}{\log\frac{x_{n}}{a}}$. Let $g_{0}\left(u_{j+1}\right)$ be the approximate value of g(u) for $u\in [u_{j},\;u_{j+1}],\;j=0,\;1,\;\cdots ,\;n-1$. Thus, we can write:

(15) $$\begin{array}{cc} & =\;\;\;\;\;\;\;\int_{0}^{1}u^{-(\alpha +1)}g_{0}\left(u\right)du\\ = & \sum_{j=0}^{n-1}=\;\;\;\;\;\;\;\int_{u_{j}}^{u_{j+1}}u^{-(\alpha +1)}g_{0}\left(u_{j+1}\right)du\\ = & =\;\;\;\;\;\;\;\int_{u_{0}}^{u_{1}}u^{-(\alpha +1)}g_{0}\left(u_{1}\right)du+\sum_{j=1}^{n-1}\int_{u_{j}}^{u_{j+1}}u^{-(\alpha +1)}g_{0}\left(u_{j+1}\right)du\\ = & -\frac{1}{\alpha }\left[g_{0}\left(u_{1}\right){\left(u_{1}-u_{0}\right)}^{-\alpha }+\sum_{j=1}^{n-1}g_{0}\left(u_{j+1}\right)u^{-\alpha }{|}_{u_{j}}^{u_{j+1}}\right]\\ = & -\frac{1}{\alpha }\left[g_{0}\left(u_{1}\right){\left(\frac{\log\frac{x_{n}}{x_{n-1}}}{\log\frac{x_{n}}{a}}\right)}^{-\alpha }+\sum_{j=1}^{n-1}g_{0}\left(u_{j+1}\right)\left({\left(\frac{\log\frac{x_{n}}{x_{n-j-1}}}{\log\frac{x_{n}}{a}}\right)}^{-\alpha }-{\left(\frac{\log\frac{x_{n}}{x_{n-j}}}{\log\frac{x_{n}}{a}}\right)}^{-\alpha }\right)\right]\\ = & -\frac{1}{\alpha }\left[f_{0}\left(x_{n-1}\right){\left(\frac{\log\frac{x_{n}}{x_{n-1}}}{\log\frac{x_{n}}{a}}\right)}^{-\alpha }+\sum_{j=1}^{n-1}f_{0}\left(x_{n-j-1}\right)\left({\left(\frac{\log\frac{x_{n}}{x_{n-j-1}}}{\log\frac{x_{n}}{a}}\right)}^{-\alpha }-{\left(\frac{\log\frac{x_{n}}{x_{n-j}}}{\log\frac{x_{n}}{a}}\right)}^{-\alpha }\right)\right]. \end{array}$$

During the calculation, we have used Lemma 3, that is,

(16) $$\begin{array}{cc} & =\;\;\;\;\;\;\;\int_{u_{0}}^{u_{1}}u^{-(\alpha +1)}g_{0}\left(u_{1}\right)du\\ = & -\frac{1}{\alpha }g_{0}\left(u_{1}\right){\left(u_{1}-u_{0}\right)}^{-\alpha }. \end{array}$$

Therefore, we get the value of the finite part integral of the Hadamard derivative at $x_{n}$:

(17) $$\begin{array}{cc} I= & \frac{1}{\Gamma (-\alpha )}{\left(\log\frac{x_{n}}{a}\right)}^{-\alpha }I_{1}\\ = & \frac{1}{\Gamma (1-\alpha )}\left[f_{0}\left(x_{n-1}\right){\left(\log\frac{x_{n}}{x_{n-1}}\right)}^{-\alpha }+\sum_{j=1}^{n-1}f_{0}\left(x_{n-j-1}\right)\left({\left(\log\frac{x_{n}}{x_{n-j-1}}\right)}^{-\alpha }-{\left(\log\frac{x_{n}}{x_{n-j}}\right)}^{-\alpha }\right)\right]\\ = & \sum_{j=0}^{n-1}\omega_{j,\;n}f_{0}\left(x_{n-j-1}\right), \end{array}$$

where $\omega_{j,\;n}$ are log-convolution coefficients given as:

(18) $$\omega_{j,\;n}=\frac{1}{\Gamma (1-\alpha )}\cdot \left\{\begin{array}{c} \;{\left(\log\frac{x_{n}}{x_{n-1}}\right)}^{-\alpha },\;j=0,\\ \;\left[{\left(\log\frac{x_{n}}{x_{n-j-1}}\right)}^{-\alpha }-{\left(\log\frac{x_{n}}{x_{n-j}}\right)}^{-\alpha }\right],\;1\leq j\leq n-1. \end{array}\right.$$

The above Scheme (17) is the left rectangular formula.

It will lead to different schemes by choosing different $g_{0}\left(u\right)$. Here, we choose two other kinds of $g_{0}\left(u\right)$ to derive the right rectangular scheme and trapezoidal formula, respectively.

(i) By choosing $g_{0}\left(u\right)$ as:
  (19) $$g_{0}\left(u\right)=g_{0}\left(u_{j}\right),\;u\in \left[u_{j},\;u_{j+1}\right],\;j=0,\;1,\;\cdots ,\;n-1,$$
  the right rectangular formula is:
  (20) $$I=\sum_{j=0}^{n-1}\omega_{j,\;n}f_{0}\left(x_{n-j}\right),$$
  where the coefficients $\omega_{j,\;n}\left(0\leq j\leq n-1\right)$ are defined as (18).
(ii) If $g_{0}\left(u\right)$ is:
  (21) $$g_{0}\left(u\right)=\frac{1}{2}\left[g_{0}\left(u_{j}\right)+g_{0}\left(u_{j+1}\right)\right],\;u\in \left[u_{j},\;u_{j+1}\right],\;j=0,\;1,\;\cdots ,\;n-1,$$
  the trapezoidal formula is given by:
  (22) $$I=\sum_{j=0}^{n}{\tilde{\omega }}_{j,\;n}f\left(x_{j}\right),$$
  in which:
  (23) $${\tilde{\omega }}_{j,\;n}=\frac{1}{2}\cdot \left\{\begin{array}{c} \;\omega_{j,\;n},\;j=0,\\ \;\left[\omega_{j-1,\;n}+\omega_{j,\;n}\right],\;1\leq j\leq n-1,\\ \;\omega_{j-1,\;n},\;j=n, \end{array}\right.$$
  where the coefficients $\omega_{j,\;n}$ are defined as (18).

By employing Lemma 4, we can directly get the following result.

**Theorem** **1.** *Suppose $f\left(x\right)\in C^{1}\left[a,\;b\right]$. For 0<α<1, the left rectangular scheme *(17)* has the estimate:*

(24) $$|\frac{1}{\Gamma (-\alpha )}=\;\;\;\;\;\;\;\int_{a}^{x_{n}}\frac{f(t)}{{\left(\log\frac{x_{n}}{t}\right)}^{\alpha +1}}\frac{dt}{t}-\sum_{j=0}^{n-1}\omega_{j,\;n}f_{0}\left(x_{n-j-1}\right)|\leq Ch^{1-\alpha },$$

*where $f_{0}\left(x_{n-j-1}\right)$ be the approximate value of f(x) for $x\in [x_{j},\;x_{j+1}],\;j=0,\;1,\;\cdots ,\;n-1$, the coefficients $\omega_{j,\;n}\left(0\leq j\leq n-1\right)$ are defined as *(18)*, and C is a constant.*

**Remark** **1.** *The estimates of the right rectangular scheme *(20)* and trapezoidal formula *(22)* are similar to the left rectangular case *(17)**

## 4. Application to the Fractional Differential Equation with the Hadamard Derivative

In the present section, we shall use the finite part integral method to solve the fractional differential equation with the Hadamard derivative.

Consider the following initial value problem:

(25) $$\left\{\begin{array}{c} H^{D_{a^{+}}^{\alpha }}u\left(x\right)=f\left(x,\;u\right),\;0<a\leq x\leq b,\;0<\alpha <1,\\ H^{D_{a^{+}}^{\alpha -1}}{u\left(x\right)\;|}_{x=a}=u_{0}, \end{array}\right.$$

where f(x,u) is a given function on [a,b]. We always assume that Equation (25) has a unique solution. This is reasonable; for example, let f(x,u) satisfy the Lipschitz condition with respect to the second variable *u*. Based on the fact that the Hadamard derivative is equivalent to the corresponding finite part integral, we can replace $H^{D_{a^{+}}^{\alpha }}u\left(x\right)$ with the finite part integral of a strong singular integral, then the initial value problem (25) can be rewritten as:

(26) $$\left\{\begin{array}{c} \;\frac{1}{\Gamma (-\alpha )}=\;\;\;\;\;\;\int_{a}^{x}\frac{u(t)}{{\left(\log\frac{x}{t}\right)}^{\alpha +1}}\frac{dt}{t}=f\left(x,\;u\right),\;0<a\leq x\leq b,\;0<\alpha <1,\\ H^{D_{a^{+}}^{\alpha -1}}{u\left(x\right)\;|}_{x=a}=u_{0}. \end{array}\right.$$

In general, we take the homogeneous initial value condition, i.e., $u\left(a\right)=u_{0}=0$. In the following, we use this kind of initial value condition. Obviously, for the left side of (26), it can be approximated by the numerical schemes developed in Section 3. Next, we just list the numerical approaches.

(i) Using Scheme (17), the initial value problem (26) with the homogeneous initial value condition is:
  (27) $$\sum_{j=0}^{n-1}\omega_{j,\;n}u_{j}=f\left(x_{n},\;u_{n}\right),$$
  where the coefficients $\omega_{j,\;n}$ are defined by (18).
  Equation (27) can be written in the following matrix form:
  (28) $$\left(\begin{array}{cccc} \omega_{0,\;1}\\ \omega_{0,\;2} & \omega_{1,\;2}\\ \vdots & \vdots & \ddots \\ \omega_{0,\;n} & \omega_{1,\;n} & \cdots & \omega_{n-1,\;n} \end{array}\right)\left(\begin{array}{c} u_{0}\\ u_{1}\\ \vdots \\ u_{n-1} \end{array}\right)=\left(\begin{array}{c} f(x_{1},\;u_{1})\\ f(x_{2},\;u_{2})\\ \vdots \\ f(x_{n},\;u_{n}) \end{array}\right).$$
(ii) Scheme (20) is used to discretize the left side of (26); we get:
  (29) $$\sum_{j=0}^{n-1}\omega_{j,\;n}u_{j+1}=f\left(x_{n},\;u_{n}\right);$$
  see (18) for more details about the coefficients $\omega_{j,\;n}$.
  The matrix form of Equation (29) is:
  (30) $$\left(\begin{array}{cccc} \omega_{0,\;1}\\ \omega_{0,\;2} & \omega_{1,\;2}\\ \vdots & \vdots & \ddots \\ \omega_{0,\;n} & \omega_{1,\;n} & \cdots & \omega_{n-1,\;n} \end{array}\right)\left(\begin{array}{c} u_{1}\\ u_{2}\\ \vdots \\ u_{n} \end{array}\right)=\left(\begin{array}{c} f(x_{1},\;u_{1})\\ f(x_{2},\;u_{2})\\ \vdots \\ f(x_{n},\;u_{n}) \end{array}\right).$$
(iii) By Scheme (22), we obtain:
  (31) $$\sum_{j=0}^{n}{\tilde{\omega }}_{j,\;n}u_{j}=f\left(x_{n},\;u_{n}\right),$$
  where ${\tilde{\omega }}_{j,\;n}$ is given by (23).
  Equation (31) can be written the matrix form as:
  (32) $$\left(\begin{array}{ccccc} {\tilde{\omega }}_{0,\;1} & {\tilde{\omega }}_{1,\;1}\\ {\tilde{\omega }}_{0,\;2} & {\tilde{\omega }}_{1,\;2} & {\tilde{\omega }}_{2,\;2}\\ \vdots & \vdots & \ddots & \ddots \\ {\tilde{\omega }}_{0,\;n} & {\tilde{\omega }}_{1,\;n} & \cdots & {\tilde{\omega }}_{n-1,\;n} & {\tilde{\omega }}_{n,\;n} \end{array}\right)\left(\begin{array}{c} u_{0}\\ u_{1}\\ \vdots \\ u_{n} \end{array}\right)=\left(\begin{array}{c} f(x_{1},\;u_{1})\\ f(x_{2},\;u_{2})\\ \vdots \\ f(x_{n},\;u_{n}) \end{array}\right).$$

Above all, we shall mention that the right-hand side function f(x,u) in the schemes (27), (29) and (31) can be presented as the form αx(t)+f(t) or the nonlinear case f(x,u(x)), where α is a constant. For the former form, it is easy to evaluate. In the latter nonlinear case, we can deal with it by combining an explicit scheme to obtain a predictor-corrector method. Here, we shall not dwell on the details in this respect.

## 5. Numerical Examples

Obviously, the Hadamard derivative is somewhat different from the Riemann–Liouville one. If f(x) can be expanded under the basis functions $1,\;x,\;x^{2},\;\cdots$, then we use its Riemann–Liouville derivative and/or integral to model the real-world problems. If f(x) can be expanded under the basis functions $1,\;\log x,\;\log^{2}x,\;\cdots$, then we use its Hadamard derivative and/or integral to describe the practical problems. Due to such special characteristics, in the present section, we first give the approximations of three basic functions to test the derived numerical schemes. Then, we use the derived numerical approximations to solve the differential equation with the Hadamard derivative.

**Example** **1.** *Suppose 0<α<1,f(x)=logx,x∈(1,2). Compute the Hadamard derivative with order α at point x=2.*

The analytical value is:

(33) $$H^{D_{1^{+}}^{\alpha }}\log x{|}_{x=2}=\frac{1}{\Gamma (2-\alpha )}{\left(\log2\right)}^{1-\alpha }.$$

Without loss of generality, we take α=0.3,0.5, respectively. We set different steps to test the fractional left rectangular formula (17), the fractional right rectangular formula (20), and the fractional trapezoidal formula (22), respectively. The numerical results are shown in Table 1, Table 2 and Table 3. We can find that the numerical results show good agreement with the analytical value.

**Example** **2.** *Suppose $0<\alpha <1,\;f\left(x\right)=\log^{2}x,\;x\in \left(1,\;2\right)$. Evaluate the Hadamard derivative with order α at point x=2.*

The analytical expression is:

(34) $$H^{D_{1^{+}}^{\alpha }}\log^{2}x{|}_{x=2}=\frac{2}{\Gamma (3-\alpha )}{\left(\log2\right)}^{2-\alpha }.$$

We also choose Schemes (17), (20) and (22) to get the approximate values. The results are included in Table 4, Table 5 and Table 6. The numerical results are in agreement with the exact solution.

**Example** **3.** *Suppose $0<\alpha <1,\;f\left(x\right)=\log^{3}x,\;x\in \left(1,\;2\right)$. Evaluate the Hadamard derivative with order α at point x=2 numerically.*

The exact value is:

(35) $$H^{D_{1^{+}}^{\alpha }}\log^{3}x{|}_{x=2}=\frac{6}{\Gamma (4-\alpha )}{\left(\log2\right)}^{3-\alpha }.$$

We also apply the three schemes (17), (20) and (22) to compute the Hadamard derivative. The results are shown in Table 7, Table 8 and Table 9, which are inline with the analytical solution.

Next, we apply the preceding schemes to solve the differential equation with the Hadamard derivative.

**Example** **4.** *Consider the following differential equation with the Hadamard derivative:*

(36) $$\left\{\begin{array}{c} H^{D_{1^{+}}^{\alpha }}u\left(x\right)=f\left(x\right),\;1\leq x\leq 2,\\ \;u(1)=0, \end{array}\right.$$

*in which 0<α<1, $f\left(x\right)=\frac{\Gamma (3-\alpha )}{\Gamma (3-2\alpha )}{\left(\log x\right)}^{2-2\alpha }$.*

The exact solution in this case is:

(37) $$\begin{array}{c} u\left(x\right)={\left(\log x\right)}^{2-\alpha }. \end{array}$$

**Figure 1:**
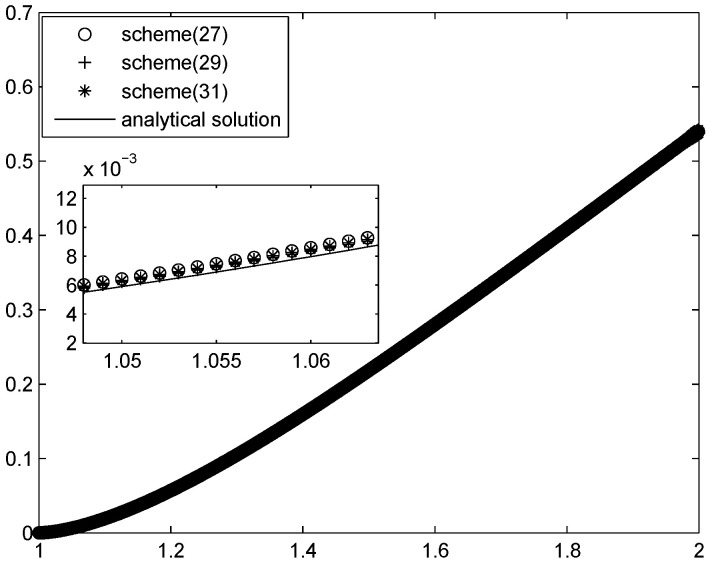
The numerical solutions of Schemes (27), (29) and (31) for Example 4, where α=0.3.

We apply Schemes (27), (29) and (31) to obtain the approximation. The errors are listed in Table 10, Table 11 and Table 12. To compare the solutions of the three schemes with the exact value, we plot the corresponding diagram; see Figure 1.

## 6. Conclusions

In this paper, we establish the numerical methods to evaluate the Hadamard derivative by employing the finite part integral and give the error analysis correspondingly. Such numerical schemes are applied to solving the fractional differential equation with the Hadamard derivative. Several numerical examples are provided to verify that the proposed numerical methods are computationally efficient. Hadamard integrals and/or derivatives emerge in the formulation of many problems in mechanics and engineering, such as the fatigue fracture of materials, so the efficient derived methods may be directly used to deal with these issues. Additionally, the approximation approaches can be applied to the analysis entropy involved in the Hadamard derivative in future research.

## Figures and Tables

**Table 1:** Approximate values in Example 1 using the left rectangular formula (17).

α	*h*	Approximate Value	Absolute Error
0.3	$\frac{1}{200}$	$8.402712\times 10^{-1}$	$1.123257\times 10^{-2}$
	$\frac{1}{2000}$	$8.493357\times 10^{-1}$	$2.168088\times 10^{-3}$
	$\frac{1}{20,000}$	$8.510782\times 10^{-1}$	$4.255374\times 10^{-4}$
	$\frac{1}{200,000}$	$8.514196\times 10^{-1}$	$8.420554\times 10^{-5}$
	$\frac{1}{400,000}$	$8.514520\times 10^{-1}$	$5.175336\times 10^{-5}$
	$\frac{1}{4,000,000}$	$8.514935\times 10^{-1}$	$1.029119\times 10^{-5}$
0.5	$\frac{1}{200}$	$8.979970\times 10^{-1}$	$4.144028\times 10^{-2}$
	$\frac{1}{2000}$	$9.263899\times 10^{-1}$	$1.304739\times 10^{-2}$
	$\frac{1}{20,000}$	$9.353158\times 10^{-1}$	$4.121460\times 10^{-3}$
	$\frac{1}{200,000}$	$9.381344\times 10^{-1}$	$1.302910\times 10^{-3}$
	$\frac{1}{400,000}$	$9.385160\times 10^{-1}$	$9.212584\times 10^{-4}$
	$\frac{1}{4,000,000}$	$9.391460\times 10^{-1}$	$2.913077\times 10^{-4}$

**Table 2:** Approximate values in Example 1 using the right rectangular formula (20).

α	*h*	Approximate Value	Absolute Error
0.3	$\frac{1}{200}$	$8.417024\times 10^{-1}$	$9.801357\times 10^{-3}$
	$\frac{1}{2000}$	$8.494767\times 10^{-1}$	$2.027073\times 10^{-3}$
	$\frac{1}{20,000}$	$8.510923\times 10^{-1}$	$4.114779\times 10^{-4}$
	$\frac{1}{200,000}$	$8.514210\times 10^{-1}$	$8.280042\times 10^{-5}$
	$\frac{1}{400,000}$	$8.514527\times 10^{-1}$	$5.105085\times 10^{-5}$
	$\frac{1}{4,000,000}$	$8.514936\times 10^{-1}$	$1.022094\times 10^{-5}$
0.5	$\frac{1}{200}$	$8.984628\times 10^{-1}$	$4.097444\times 10^{-2}$
	$\frac{1}{2000}$	$9.264294\times 10^{-1}$	$1.300785\times 10^{-2}$
	$\frac{1}{20,000}$	$9.353195\times 10^{-1}$	$4.117730\times 10^{-3}$
	$\frac{1}{200,000}$	$9.381347\times 10^{-1}$	$1.302544\times 10^{-3}$
	$\frac{1}{400,000}$	$9.385162\times 10^{-1}$	$9.210758\times 10^{-4}$
	$\frac{1}{4,000,000}$	$9.391460\times 10^{-1}$	$2.912895\times 10^{-4}$

**Table 3:** Approximate values in Example 1 using the trapezoidal formula (22).

α	*h*	Approximate Value	Absolute Error
0.3	$\frac{1}{200}$	$8.409868\times 10^{-1}$	$1.051696\times 10^{-2}$
	$\frac{1}{2000}$	$8.494062\times 10^{-1}$	$2.097581\times 10^{-3}$
	$\frac{1}{20,000}$	$8.510853\times 10^{-1}$	$4.185077\times 10^{-4}$
	$\frac{1}{200,000}$	$8.514203\times 10^{-1}$	$8.350298\times 10^{-5}$
	$\frac{1}{400,000}$	$8.514524\times 10^{-1}$	$5.140210\times 10^{-5}$
	$\frac{1}{4,000,000}$	$8.514935\times 10^{-1}$	$1.025607\times 10^{-5}$
0.5	$\frac{1}{200}$	$8.982299\times 10^{-1}$	$4.120736\times 10^{-2}$
	$\frac{1}{2000}$	$9.264097\times 10^{-1}$	$1.302762\times 10^{-2}$
	$\frac{1}{20,000}$	$9.353177\times 10^{-1}$	$4.119595\times 10^{-3}$
	$\frac{1}{200,000}$	$9.381346\times 10^{-1}$	$1.302727\times 10^{-3}$
	$\frac{1}{400,000}$	$9.385161\times 10^{-1}$	$9.211671\times 10^{-4}$
	$\frac{1}{4,000,000}$	$9.391460\times 10^{-1}$	$2.912986\times 10^{-4}$

**Table 4:** Approximate values in Example 2 using the left rectangular formula (17).

α	*h*	Approximate Value	Absolute Error
0.3	$\frac{1}{200}$	$6.779686\times 10^{-1}$	$1.640482\times 10^{-2}$
	$\frac{1}{2000}$	$6.912817\times 10^{-1}$	$3.091752\times 10^{-3}$
	$\frac{1}{20,000}$	$6.937749\times 10^{-1}$	$5.985904\times 10^{-4}$
	$\frac{1}{200,000}$	$6.942559\times 10^{-1}$	$1.176018\times 10^{-4}$
	$\frac{1}{400,000}$	$6.943013\times 10^{-1}$	$7.217952\times 10^{-5}$
	$\frac{1}{4,000,000}$	$6.943591\times 10^{-1}$	$1.431004\times 10^{-5}$
0.5	$\frac{1}{200}$	$8.093944\times 10^{-1}$	$5.882996\times 10^{-2}$
	$\frac{1}{2000}$	$8.499898\times 10^{-1}$	$1.823463\times 10^{-2}$
	$\frac{1}{20,000}$	$8.624958\times 10^{-1}$	$5.728553\times 10^{-3}$
	$\frac{1}{200,000}$	$8.664167\times 10^{-1}$	$1.807726\times 10^{-3}$
	$\frac{1}{400,000}$	$8.669465\times 10^{-1}$	$1.277890\times 10^{-3}$
	$\frac{1}{4,000,000}$	$8.678205\times 10^{-1}$	$4.039138\times 10^{-4}$

**Table 5:** Approximate values in Example 2 using the right rectangular formula (20).

α	*h*	Approximate Value	Absolute Error
0.3	$\frac{1}{200}$	$6.816369\times 10^{-1}$	$1.273655\times 10^{-2}$
	$\frac{1}{2000}$	$6.916498\times 10^{-1}$	$2.723630\times 10^{-3}$
	$\frac{1}{20,000}$	$6.938117\times 10^{-1}$	$5.617524\times 10^{-4}$
	$\frac{1}{200,000}$	$6.942595\times 10^{-1}$	$1.139175\times 10^{-4}$
	$\frac{1}{400,000}$	$6.943031\times 10^{-1}$	$7.033734\times 10^{-5}$
	$\frac{1}{4,000,000}$	$6.943593\times 10^{-1}$	$1.412582\times 10^{-5}$
0.5	$\frac{1}{200}$	$8.093944\times 10^{-1}$	$5.882996\times 10^{-2}$
	$\frac{1}{2000}$	$8.499898\times 10^{-1}$	$1.823463\times 10^{-2}$
	$\frac{1}{20,000}$	$8.624958\times 10^{-1}$	$5.728553\times 10^{-3}$
	$\frac{1}{200,000}$	$8.664167\times 10^{-1}$	$1.807726\times 10^{-3}$
	$\frac{1}{400,000}$	$8.669465\times 10^{-1}$	$1.277890\times 10^{-3}$
	$\frac{1}{4,000,000}$	$8.678205\times 10^{-1}$	$4.039138\times 10^{-4}$

**Table 6:** Approximate values in Example 2 using the trapezoidal formula (22).

α	*h*	Approximate Value	Absolute Error
0.3	$\frac{1}{200}$	$6.798025\times 10^{-1}$	$1.457095\times 10^{-2}$
	$\frac{1}{2000}$	$6.914658\times 10^{-1}$	$2.907693\times 10^{-3}$
	$\frac{1}{20,000}$	$6.937933\times 10^{-1}$	$5.801714\times 10^{-4}$
	$\frac{1}{200,000}$	$6.942577\times 10^{-1}$	$1.157596\times 10^{-4}$
	$\frac{1}{400,000}$	$6.943022\times 10^{-1}$	$7.125843\times 10^{-5}$
	$\frac{1}{4,000,000}$	$6.943592\times 10^{-1}$	$1.421793\times 10^{-5}$
0.5	$\frac{1}{200}$	$8.111277\times 10^{-1}$	$5.709665\times 10^{-2}$
	$\frac{1}{2000}$	$8.501652\times 10^{-1}$	$1.805920\times 10^{-2}$
	$\frac{1}{20,000}$	$8.625135\times 10^{-1}$	$5.710942\times 10^{-3}$
	$\frac{1}{200,000}$	$8.664184\times 10^{-1}$	$1.805962\times 10^{-3}$
	$\frac{1}{400,000}$	$8.669474\times 10^{-1}$	$1.277008\times 10^{-3}$
	$\frac{1}{4,000,000}$	$8.678206\times 10^{-1}$	$4.038256\times 10^{-4}$

**Table 7:** Approximate values in Example 3 using the left rectangular formula (17).

α	*h*	Approximate Value	Absolute Error
0.3	$\frac{1}{200}$	$5.172901\times 10^{-1}$	$1.749097\times 10^{-2}$
	$\frac{1}{2000}$	$5.315199\times 10^{-1}$	$3.261170\times 10^{-3}$
	$\frac{1}{20,000}$	$5.341540\times 10^{-1}$	$6.270883\times 10^{-4}$
	$\frac{1}{200,000}$	$5.346584\times 10^{-1}$	$1.227464\times 10^{-4}$
	$\frac{1}{400,000}$	$5.347058\times 10^{-1}$	$7.528326\times 10^{-5}$
	$\frac{1}{4,000,000}$	$5.347662\times 10^{-1}$	$1.490212\times 10^{-5}$
0.5	$\frac{1}{200}$	$6.601966\times 10^{-1}$	$6.197217\times 10^{-2}$
	$\frac{1}{2000}$	$7.031196\times 10^{-1}$	$1.904914\times 10^{-2}$
	$\frac{1}{20,000}$	$7.162033\times 10^{-1}$	$5.965417\times 10^{-3}$
	$\frac{1}{200,000}$	$7.202883\times 10^{-1}$	$1.880472\times 10^{-3}$
	$\frac{1}{400,000}$	$7.208396\times 10^{-1}$	$1.329120\times 10^{-3}$
	$\frac{1}{4,000,000}$	$7.217488\times 10^{-1}$	$4.200048\times 10^{-4}$

**Table 8:** Approximate values in Example 3 using the right rectangular formula (20).

α	*h*	Approximate Value	Absolute Error
0.3	$\frac{1}{200}$	$5.219967\times 10^{-1}$	$1.278441\times 10^{-2}$
	$\frac{1}{2000}$	$5.319963\times 10^{-1}$	$2.784793\times 10^{-3}$
	$\frac{1}{20,000}$	$5.342018\times 10^{-1}$	$5.793365\times 10^{-4}$
	$\frac{1}{200,000}$	$5.346631\times 10^{-1}$	$1.179689\times 10^{-4}$
	$\frac{1}{400,000}$	$5.347082\times 10^{-1}$	$7.289443\times 10^{-5}$
	$\frac{1}{4,000,000}$	$5.347665\times 10^{-1}$	$1.466322\times 10^{-5}$
0.5	$\frac{1}{200}$	$6.601966\times 10^{-1}$	$6.197217\times 10^{-2}$
	$\frac{1}{2000}$	$7.031196\times 10^{-1}$	$1.904914\times 10^{-2}$
	$\frac{1}{20,000}$	$7.162033\times 10^{-1}$	$5.965417\times 10^{-3}$
	$\frac{1}{200,000}$	$7.202883\times 10^{-1}$	$1.880472\times 10^{-3}$
	$\frac{1}{400,000}$	$7.208396\times 10^{-1}$	$1.329120\times 10^{-3}$
	$\frac{1}{4,000,000}$	$7.217488\times 10^{-1}$	$4.200048\times 10^{-4}$

**Table 9:** Approximate values in Example 3 using the trapezoidal formula (22).

α	*h*	Approximate Value	Absolute Error
0.3	$\frac{1}{200}$	$5.196406\times 10^{-1}$	$1.514051\times 10^{-2}$
	$\frac{1}{2000}$	$5.317581\times 10^{-1}$	$3.023009\times 10^{-3}$
	$\frac{1}{20,000}$	$5.341779\times 10^{-1}$	$6.032127\times 10^{-4}$
	$\frac{1}{200,000}$	$5.346608\times 10^{-1}$	$1.203576\times 10^{-4}$
	$\frac{1}{400,000}$	$5.347070\times 10^{-1}$	$7.408885\times 10^{-5}$
	$\frac{1}{4,000,000}$	$5.347663\times 10^{-1}$	$1.478267\times 10^{-5}$
0.5	$\frac{1}{200}$	$6.628348\times 10^{-1}$	$5.933399\times 10^{-2}$
	$\frac{1}{2000}$	$7.033932\times 10^{-1}$	$1.877556\times 10^{-2}$
	$\frac{1}{20,000}$	$7.162310\times 10^{-1}$	$5.937755\times 10^{-3}$
	$\frac{1}{200,000}$	$7.202911\times 10^{-1}$	$1.877696\times 10^{-3}$
	$\frac{1}{400,000}$	$7.208410\times 10^{-1}$	$1.327732\times 10^{-3}$
	$\frac{1}{4,000,000}$	$7.217489\times 10^{-1}$	$4.198658\times 10^{-4}$

**Table 10:** The absolute errors for Equation (36) at x=2 using Scheme (27).

α	*h*	Absolute Error	α	*h*	Absolute Error
0.1	$\frac{1}{80}$	$7.965781\times 10^{-2}$	0.3	$\frac{1}{80}$	$1.789693\times 10^{-1}$
	$\frac{1}{160}$	$5.873791\times 10^{-2}$		$\frac{1}{160}$	$1.488886\times 10^{-1}$
	$\frac{1}{320}$	$4.339783\times 10^{-2}$		$\frac{1}{320}$	$1.250382\times 10^{-1}$
	$\frac{1}{640}$	$3.211236\times 10^{-2}$		$\frac{1}{640}$	$1.057895\times 10^{-1}$
	$\frac{1}{1280}$	$2.379473\times 10^{-2}$		$\frac{1}{1280}$	$9.001701\times 10^{-2}$
	$\frac{1}{2560}$	$1.765154\times 10^{-2}$		$\frac{1}{2560}$	$7.694314\times 10^{-2}$

**Table 11:** The absolute errors for Equation (36) at x=2 using Scheme (29).

α	*h*	Absolute Error	α	*h*	Absolute Error
0.1	$\frac{1}{80}$	$2.062341\times 10^{-2}$	0.3	$\frac{1}{80}$	$1.175531\times 10^{-1}$
	$\frac{1}{160}$	$1.699482\times 10^{-2}$		$\frac{1}{160}$	$1.054987\times 10^{-1}$
	$\frac{1}{320}$	$1.388033\times 10^{-2}$		$\frac{1}{320}$	$9.437406\times 10^{-2}$
	$\frac{1}{640}$	$1.123930\times 10^{-2}$		$\frac{1}{640}$	$8.411439\times 10^{-2}$
	$\frac{1}{1280}$	$9.034460\times 10^{-3}$		$\frac{1}{1280}$	$7.469366\times 10^{-2}$
	$\frac{1}{2560}$	$7.213855\times 10^{-3}$		$\frac{1}{2560}$	$6.610924\times 10^{-2}$

**Table 12:** The absolute errors for Equation (36) at x=2 using Scheme (31).

α	*h*	Absolute Error	α	*h*	Absolute Error
0.1	$\frac{1}{80}$	$5.048797\times 10^{-2}$	0.3	$\frac{1}{80}$	$1.494174\times 10^{-1}$
	$\frac{1}{160}$	$3.810241\times 10^{-2}$		$\frac{1}{160}$	$1.279494\times 10^{-1}$
	$\frac{1}{320}$	$2.888364\times 10^{-2}$		$\frac{1}{320}$	$1.103626\times 10^{-1}$
	$\frac{1}{640}$	$2.198710\times 10^{-2}$		$\frac{1}{640}$	$9.563445\times 10^{-2}$
	$\frac{1}{1280}$	$1.682600\times 10^{-2}$		$\frac{1}{1280}$	$8.303033\times 10^{-2}$
	$\frac{1}{2560}$	$1.297695\times 10^{-2}$		$\frac{1}{2560}$	$6.532645\times 10^{-2}$

## References

[1] Kilbas A.A., Srivastava H.M., Trujillo J.J. (2006). Theory and Applications of Fractional Differential Equations.

[2] Podlubny I. (1999). Fractional Differential Equations.

[3] Li C.P., Zeng F.H. (2015). Numerical Methods for Fractional Calculus.

[4] Xu K.X., Wang J. (2017). Weighted fractional permutation entropy and fractional sample entropy for nonlinear Potts financial dynamics. Phys. Lett. A.

[5] Machado J.T. (2014). Fractional order generalized information. Entropy.

[6] Munoz-Pacheco J.M., Zambrano-Serrano E., Volos C., Jafari C., Kengne J., Rajagopal K. (2018). A new fractional-order chaotic system with different families of hidden and self-excited attractors. Entropy.

[7] Goldfain E. (2008). Fractional dynamics and the Standard Model for particle physics. Commun. Nonlinear Sci. Numer. Simul..

[8] Kulish V.V., Lage J.L. (2002). Application of fractional calculus to fluid mechanics. J. Fluids Eng.-Trans. ASME.

[9] Ma L., Li C.P. (2016). Center manifold of fractional dynamical system. J. Comput. Nonlinear Dyn..

[10] Ionescu C.M. (2013). Emerging tools in engineering: Fractional order Ladder Impedance Models for respiratory and neural Systems. IEEE J. Emerg. Sel. Top. Circuits Syst..

[11] Scalas E., Gorenflo R., Mainardi F. (2000). Fractional calculus and continuous-time finance. Phys. A.

[12] Magin R.L. (2010). Fractional calculus models of complex dynamics in biological tissues. Comput. Math. Appl..

[13] Li C.P., Yi Q., Kurths J. (2018). Fractional convection. J. Comput. Nonlinear Dyn..

[14] Arshad S., Baleanu D., Huang J.F., Al Qurashi M.M., Tang Y., Zhao Y. (2018). Finite difference method for time-space fractional advection-diffusion equations with Riesz derivative. Entropy.

[15] Hadamard J. (1892). Essai sur létude des fonctions données par leur développment de Taylor. J. Math. Pures Appl. Ser..

[16] Kilbas A.A., Trujillo J.J. (2003). Hadamard-type integrals as G-transforms. Integral Transform. Spec. Funct..

[17] Kilbas A.A. (2001). Hadamard-type fractional calculus. J. Korean Math. Soc..

[18] Butzer P.L., Kilbas A.A., Trujillo J.J. (2002). Fractional calculus in the Mellin setting and Hadamard-type fractional integrals. J. Math. Anal. Appl..

[19] Ma L., Li C.P. (2017). On Hadamard fractional calculus. Fractals.

[20] Butzer P.L., Kilbas A.A., Trujillo J.J. (2002). Mellin transform analysis and integration by parts for Hadamard-type fractional integrals. J. Math. Anal. Appl..

[21] Jarad F., Abdeljawad T., Baleanu D. (2012). Caputo-type modification of the Hadamard fractional derivatives. Adv. Differ. Equ..

[22] Kamocki R. (2015). Necessary and sufficient conditions for the existence of the Hadamard-type fractional derivative. Integral Transform. Spec. Funct..

[23] Pooseh S., Almeida R., Torres D.F.M. (2012). Expansion formulas in terms of integer-order derivatives for the Hadamard fractional integral and derivative. Numer. Funct. Anal. Optim..

[24] He S.B., Sun K.H., Wang R.X. (2018). Fractional fuzzy entropy algorithm and the complexity analysis for nonlinear time series. Eur. Phys. J. Spec. Top..

[25] Lopes A.M., Machado J.A.T. (2016). Integer and fractional-order entropy analysis of earthquake data series. Nonlinear Dyn..

[26] Garra R., Orsingher E., Polito F. (2018). A note on Hadamard fractional differential equations with varying coefficients and their applications in probability. Mathematics.

[27] Diethelm K. (1997). An algorithm for the numerical solution of differential equations of fractional order. Electron. Trans. Numer. Anal..

[28] Ma L., Li C.P. (2018). On finite part integrals and Hadamard-type fractional derivatives. J. Comput. Nonlinear Dyn..

[29] Diethelm K. (1997). Generalized compound quadrature formulae for finite-part integrals. IMA J. Numer. Anal..

[30] Yin C.T., Li C.P., Bi Q.S. Approximating Hadamard derivative and fractional differential equation via the finite part integral. Proceedings of the International Conference on Fractional Differentiation and its Application (ICFDA 2018).

[31] Ioakimidis N.I. (1982). Application of finite-part integrals to the singular integral equations of crack problems in plane and three-dimensional elasticity. Acta Mech..

[32] Elliott D. (1995). Three algorithms for Hadamard finite-part integrals and fractional derivatives. J. Comput. Appl. Math..

